# Mechanisms of Vascular Dysfunction in COPD and Effects of a Novel Soluble Epoxide Hydrolase Inhibitor in Smokers

**DOI:** 10.1016/j.chest.2016.10.058

**Published:** 2017-03

**Authors:** Lucy Yang, Joseph Cheriyan, David D. Gutterman, Ruth J. Mayer, Zsuzsanna Ament, Jules L. Griffin, Aili L. Lazaar, David E. Newby, Ruth Tal-Singer, Ian B. Wilkinson

**Affiliations:** a Experimental Medicine and Immunotherapeutics (EMIT), University of Cambridge, Addenbrooke’s Hospital, Cambridge, England; b Cambridge Clinical Trials Unit, Cambridge University Hospitals NHS Foundation Trust, Cambridge, England; c Clinical Unit Cambridge, GSK R&D, Cambridge, England; d MRC Human Nutrition Research, Elsie Widdowson Laboratory; and Department of Biochemistry, University of Cambridge, Cambridge, England; e Department of Medicine, Cardiovascular Center, Medical College of Wisconsin, Milwaukee, WI; f GSK R&D, King of Prussia, PA; g British Heart Foundation Centre for Cardiovascular Science, University of Edinburgh, Edinburgh, Scotland

**Keywords:** clinical trial, COPD, EETs, endothelial function, smokers, soluble epoxide hydrolase inhibitor, CYP, cytochrome P450, DHET, dihydroxyepoxyeicosatrienoic acid, EC50, half maximal effective concentration, EDHF, endothelium-derived hyperpolarizing factor, EET, epoxyeicosatrienoic acid, LNAME, L-nitroarginine methyl ester, LNMMA, NG-monomethyl-L-arginine, NFκB, nuclear factor kappa light-chain enhancer of activated B cells, NO, nitric oxide, PAI-1, plasminogen activator inhibitor type 1, sEH, soluble epoxide hydrolase, SNP, sodium nitroprusside, tPA, tissue plasminogen activator

## Abstract

**Background:**

Smoking and COPD are risk factors for cardiovascular disease, and the pathogenesis may involve endothelial dysfunction. We tested the hypothesis that endothelium-derived epoxyeicosatrienoic acid (EET)-mediated endothelial function is impaired in patients with COPD and that a novel soluble epoxide hydrolase inhibitor, GSK2256294, attenuates EET-mediated endothelial dysfunction in human resistance vessels both in vitro and in vivo.

**Methods:**

Endogenous and stimulated endothelial release of EETs was assessed in 12 patients with COPD, 11 overweight smokers, and two matched control groups, using forearm plethysmography with intraarterial infusions of fluconazole, bradykinin, and the combination. The effects of GSK2256294 on EET-mediated vasodilation in human resistance arteries were assessed in vitro and in vivo in a phase I clinical trial in healthy overweight smokers.

**Results:**

Compared with control groups, there was reduced vasodilation with bradykinin (*P* = .005), a blunted effect of fluconazole on bradykinin-induced vasodilation (*P* = .03), and a trend toward reduced basal EET/dihydroxyepoxyeicosatrienoic acid ratio in patients with COPD (*P* = .08). A similar pattern was observed in overweight smokers. In vitro, 10 μM GSK2256294 increased 11,12-EET-mediated vasodilation compared with vehicle (90% ± 4.2% vs 72.6% ± 6.2% maximal dilatation) and shifted the bradykinin half-maximal effective concentration (EC50) (–8.33 ± 0.172 logM vs –8.10 ± 0.118 logM; *P* = .001 for EC50). In vivo, 18 mg GSK2256294 improved the maximum bradykinin response from 338% ± 46% before a dose to 566% ± 110% after a single dose (*P* = .02) and to 503% ± 123% after a chronic dose (*P* = .003).

**Conclusions:**

GSK2256294 attenuates smoking-related EET-mediated endothelial dysfunction, suggesting potential therapeutic benefits in patients with COPD.

**Trial Registry:**

ClinicalTrials.gov; No.: NCT01762774; URL: www.clinicaltrials.gov

COPD is the third leading cause of death worldwide and a risk predictor for atherosclerosis.^1^, ^2^, ^3^ Several pathophysiological processes may contribute to disease progression and increased cardiovascular risk in COPD, including systemic effects of smoking, chronic inflammation,^4^ and endothelial dysfunction.^5^ Patients with COPD are also more likely to have other cardiovascular comorbidities, including central abdominal obesity and metabolic syndrome, particularly in earlier stages of COPD.^6^, ^7^, ^8^ Endothelium-derived hyperpolarizing factor (EDHF) and, particularly, epoxyeicosatrienoic acid (EET) are involved in the modulation of vascular tone,^9^ attenuation of inflammation,^10^ and activation of fibrinolysis by augmenting tissue plasminogen activator (tPA) expression.^11^

EETs are synthesized by cytochrome P450 (CYP) enzymes, and metabolized to their less biologically active diols by soluble epoxide hydrolase (sEH) enzymes.^12^ Smoking has a synergistic effect with CYP450 and sEH polymorphisms,^13^ resulting in enhanced sEH activity, reduced plasma EETs, and increasing overall risk of myocardial infarction.^14^ Plasma EET levels are reduced in patients with coronary artery disease who are obese or who smoke.^15^ EETs are also produced in lung epithelial cells, and they may become dysfunctional in COPD.^12^ In vivo, smokers exhibit reduced endothelial responses to bradykinin,^5^ and this may be associated with impaired EDHF-mediated vasodilation.^16^, ^17^ However, the functional role of EETs has not yet been characterized in humans.

Upregulation of EETs by sEH inhibition in animals improves metabolic syndrome^18^ and lung function and attenuates smoking-related inflammation and emphysema.^19^ GSK2256294 is a novel potent sEH inhibitor in phase I clinical development and may have the potential to impact systemic and pulmonary endothelial function. As this was a phase I clinical trial mainly focused on safety and tolerability in healthy people, we used a cohort of overweight smokers as representative of patients with early-stage COPD.

We hypothesized that EET synthesis is reduced in patients with COPD and otherwise healthy overweight smokers and that sEH inhibition would upregulate EETs and endothelial dysfunction. We completed a physiological study in which we assessed EET-mediated basal tone, and the EET component of bradykinin stimulated vasodilation in patients with COPD and in overweight smokers to maximize the impact of cardiovascular risk factors in otherwise healthy subjects. Subsequently, we examined the effects of a novel sEH inhibitor, GSK2256294, in human resistance arteries in vitro and in vivo in a phase I clinical trial with an experimental medicine arm to provide early proof of mechanism for target engagement in overweight smokers. The study design, safety, and pharmacokinetic data from the phase I trial were reported separately,^20^ and we only report the effects of sEH inhibition on endothelial function in this manuscript.

## Methods

All study procedures were conducted in accordance with the Declaration of Helsinki, were approved by appropriate institutional review boards, and received favorable opinions from local ethics committees (13/EE/0032, 12/LO/1832), and the Medicines and Healthcare products Regulatory Agency. Analysis and statistical methods are described in e-Appendix 1. All subjects were recruited following written consent.

We used forearm venous occlusion plethysmography^21^ to assess vascular function in vivo with intraarterial infusion of challenge agents through a 27-gauge needle (Coopers Needleworks) inserted into the brachial artery. Venous plasma concentrations of EET/DHET were assessed as representative of sEH activity at baseline and during the forearm blood flow studies. Oscillometric BP was monitored in the noninfused arm. Detailed methods and statistical analyses can be seen in e-Appendix 1.

### Study 1

Twelve male patients with COPD (FEV_1_/FVC < 0.7 and FEV_1_ < 80% postbronchodilator use), and 12 healthy sex-matched control groups (matched control group 1) underwent a single forearm blood flow study to assess EET-mediated vasodilation (UK Clinical Research Network Portfolio ID: 14339). Patients taking concomitant medications that interfere with CYP450 or cyclooxygenase enzymes were asked to stop for at least 4 days prior to the forearm blood flow procedure. Overall endothelium-dependent function was assessed by infusing bradykinin (100, 300, and 1,000 pmol/min; Bachem Distribution Services GmbH), and stimulated EET release was assessed by coinfusing bradykinin with 0.4 μmol/min fluconazole, a CYP inhibitor that inhibits EET synthesis (Pfizer Ltd.) (e-Fig 1).^9^ Endothelium-independent responses were assessed using 12 and 38 nmol/min (3 and 10 μg/min) sodium nitroprusside (SNP) (Nitroprussiat FIDES).

### Study 2

Twelve overweight smokers (≥ 10 cigarettes/d and > 5 pack-year history, weight > 60 kg, and BMI 28-35 kg/m^2^) and equal numbers of healthy sex- and age-matched nonsmoker control groups (matched control group 2) underwent the same forearm blood flow protocol as did subjects in study 1.

### Study 3

We first assessed the effects of sEH inhibition in vitro by application of GSK2256294 to human resistance arteries treated with L-nitroarginine methyl ester (LNAME) and indomethacin (detailed description of methods in e-Appendix 1) and in vivo using forearm blood flow before a dose, after a single dose (acute effects), and after 14 days (chronic effects) of oral GSK2256294. Responses to bradykinin (300, 600, and 1,000 pmol/min) were assessed in the presence of 8 μmol/min NG-monomethyl-L-arginine (LNMMA; Bachem) and 6 mmol (1 g) IV aspirin (Aspergic Sanofi-Aventis) to inhibit nitric oxide (NO) and prostaglandin I_2_ synthesis to maximize EDHF and EET (e-Fig 2). Venous concentrations of tPA and plasminogen activator inhibitor type 1 (PAI-1) were measured before and after each dose of bradykinin.^22^ Challenge agent doses were chosen based on previous studies.^5^

To assess the effects of GSK2256294 in vivo, we studied healthy overweight smokers (no concomitant medications) as a paradigm for a COPD population in a phase I clinical trial to provide early proof of mechanism (ClinicalTrials.gov NCT01762774). Thirty male overweight smokers, were allocated in a 2:1 ratio between GSK2256294 (6 mg or 18 mg) and placebo for 14 days of repeated doses. GSK2256294 doses were chosen based on enzyme inhibition and pharmacokinetic data from the single-dosing cohorts.^20^

## Results

### Study 1

**Figure 1:**
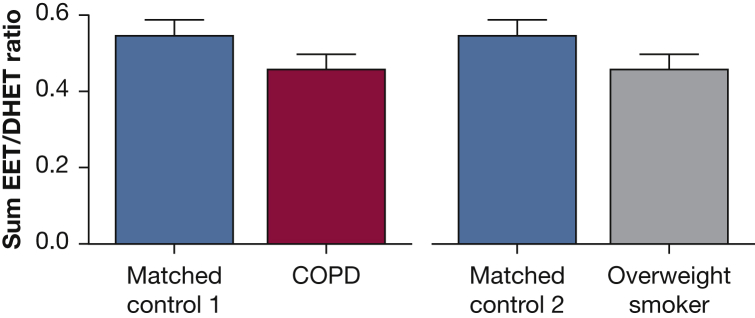
Plasma concentration of basal EET/DHET in patients with COPD and overweight smokers. There was a trend toward a higher EET/DHET ratio in matched control group 1 (blue) than in patients with COPD (red; P = .08) and a higher EET/DHET ratio in matched control group 2 (blue) compared with overweight smokers (gray; not significant). DHET = dihydroxyepoxyeicosatrienoic acid; EET = epoxyeicosatrienoic acid.

Subject demographics are presented in Table 1. The average FEV_1_ was 53% ± 13% predicted and the FEV_1_/FVC ratio was 0.5 ± 0.1 in the subjects with COPD. There was a trend toward a higher plasma concentration of the basal EET/DHET ratio in the matched control group 1 compared with patients with COPD (0.54 ± 0.12 vs 0.45 ± 0.14; *P* = .08) (Fig 1).

**Figure 2:**
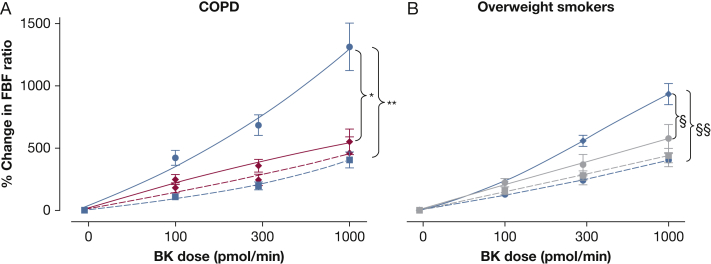
Forearm blood flow responses in (A) patients with COPD and (B) overweight smokers. Bradykinin-induced vasodilation (solid lines) was greater in healthy matched control groups (blue) than in patients with COPD (red; *P = .005) and overweight smokers (gray; ^§^P = .02). In the presence of fluconazole (dotted lines), bradykinin-induced vasodilation was reduced in healthy matched control subjects (**P < .0001 and ^§§^P < .0001) but not in patients with COPD or overweight smokers. BK = bradykinin; FBF = forearm blood flow.

There was a dose-dependent increase in the forearm blood flow ratio following bradykinin in both groups (*P* < .0001). Bradykinin response was significantly higher in the matched control group 1 than in patients with COPD (maximal dilatation 1,314% ± 191% vs 552% ± 103%; *P* = .005) (Fig 2A). In the presence of fluconazole, maximum dilatation to bradykinin was reduced in matched control group 1 (406% ± 64%; *P* < .0001) but not in patients with COPD (447% ± 124%; *P* = .32), showing a significant between-group difference in inhibition (*P* = .03). There was no difference in SNP response between groups (data not shown). BP values remained constant throughout the studies.

**Figure 3:**
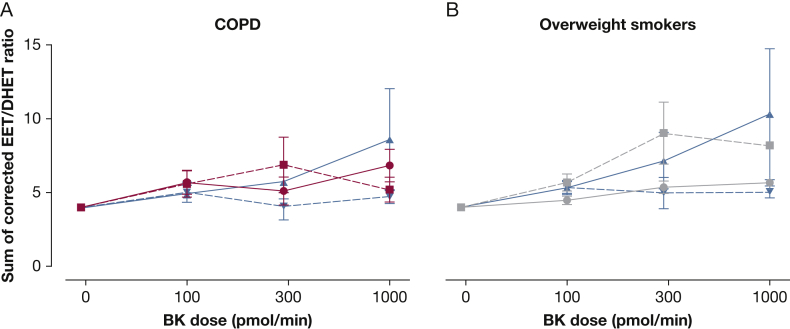
Sum of baseline corrected EET/DHET ratio in response to bradykinin in patients with (A) COPD and (B) overweight smokers. Although not significant, there was a trend toward a greater increase in total EET/DHET ratio in response to bradykinin (solid lines) in healthy subjects (blue) compared with patients with COPD (red) and overweight smokers (gray). In the presence of fluconazole (dotted lines), there was a trend toward a reduced total EET/DHET ratio in the healthy group but not in patients with COPD or overweight smokers. See Figure 1 and 2 legends for expansion of abbreviations.

Although not significant, plasma concentrations of the EET/DHET ratio in response to bradykinin was higher in the matched control group 1 compared with patients with COPD (maximum 8.6 ± 3.4 vs 6.8 ± 1.1; *P* = .83). Although it was not significant, in the presence of fluconazole, total EET/DHET levels were slightly lower in matched control group 1 (maximum 4.7 ± 0.4; *P* = .27) but not in patients with COPD (5.2 ± 0.9; *P* = .70) (Fig 3A).

### Study 2

Although not significant, the basal EET/DHET ratio was higher in the matched control group 2 compared with overweight smokers (0.46 ± 0.06 vs 0.39 ± 0.04; *P* = .33) (Fig 1).

Bradykinin response was higher in the matched control group 2 than in overweight smokers (maximal dilatation: 930% ± 81% vs 575% ± 112%; *P* = .02) (Fig 2B). In the presence of fluconazole, maximum dilatation to bradykinin was reduced in the matched control group 2 (400% ± 49%; *P* < .0001) but not in overweight smokers (437% ± 57%; *P* = .16), resulting in a significant between-group difference (*P* = .002). There was no difference in SNP response between groups (data not shown). BP values remained constant throughout the studies. There was no difference in the bradykinin response between subjects with COPD and overweight smokers (*P* = .72).

Although not significant, the increase in the EET/DHET ratio in response to bradykinin was higher in the healthy matched control group 2 compared with overweight smokers (maximum 10.31 ± 4.43 vs 5.66 ± 0.46; *P* = .80). In the presence of fluconazole, EET/DHET was reduced in the matched control group 2 but were slightly increased in overweight smokers (maximum, 5.02 ± 0.38 vs 8.19 ± 2.18; *P* = .003) (Fig 3B).

### Study 3

**Figure 4:**
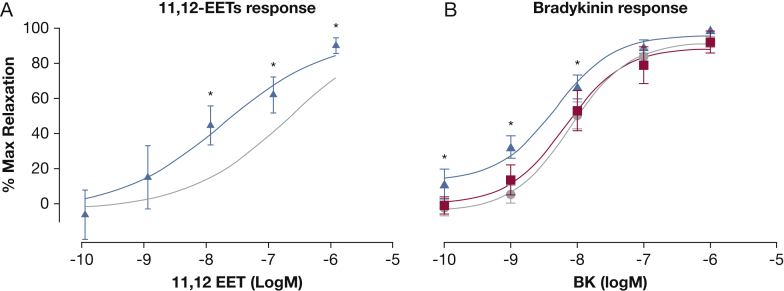
In vitro study. Effect of GSK2256294 on (A) 11,12-EET-induced vasodilation and (B) BK-induced vasodilation in LNAME- and indomethacin-treated human resistance arteries. (A) Isolated human arterioles (n = 6) were preconstricted with endothelin-1, and 11,12-EET-induced dilatation was examined in the absence and presence of 10 μM GSK2256294 (blue). (B) Bradykinin-induced dilatation was examined in the absence (gray) and presence of 1 μM (red) and 10 μM (blue) GSK2256294. *P < .05 compared with control group. LNAME = L-nitroarginine methyl ester. See Figure 1 and 2 legends for expansion of other abbreviations.

In LNAME- and indomethacin-treated resistance vessels, GSK2256294 10 μM increased 11,12-EET-mediated vasodilation compared with vehicle (n = 6 in each group; 90% ± 4% vs 73% ± 6% maximal dilatation) (Fig 4A) and shifted the bradykinin EC50 (n = 6; –8.33 ± 0.17 logM vs –8.10 ± 0.12 logM; *P* = .001) (Fig 4B). However, vasodilation from 8,9-EET was unaltered (maximal dilatation, 82% ± 16% vs 72% ± 19%), suggesting that the effects were regioisomer specific. The vasodilation from papaverine (100 μM), a test of direct smooth muscle vasodilation, was unchanged with GSK2256294 administration.

In vivo, 28 subjects, including the 11 who took part in the physiological study, completed forearm blood flow studies before dosing, after a single dose, and after 14 days of repeated dosing with placebo (n = 6) or GSK2256294, 6 mg or 18 mg (n = 11 in each group) (Table 1). There was a trend toward increased bradykinin response after single and repeated dosing in the active treatment groups. In subjects who received 6 mg, response to bradykinin increased by 23% ± 17% on day 1 and by 22% ± 22% on day 14. In those who received 18 mg, bradykinin response increased by 14% ± 17% on day 1 and 12% ± 14% on day 14. Responses to SNP did not change.

**Figure 5:**
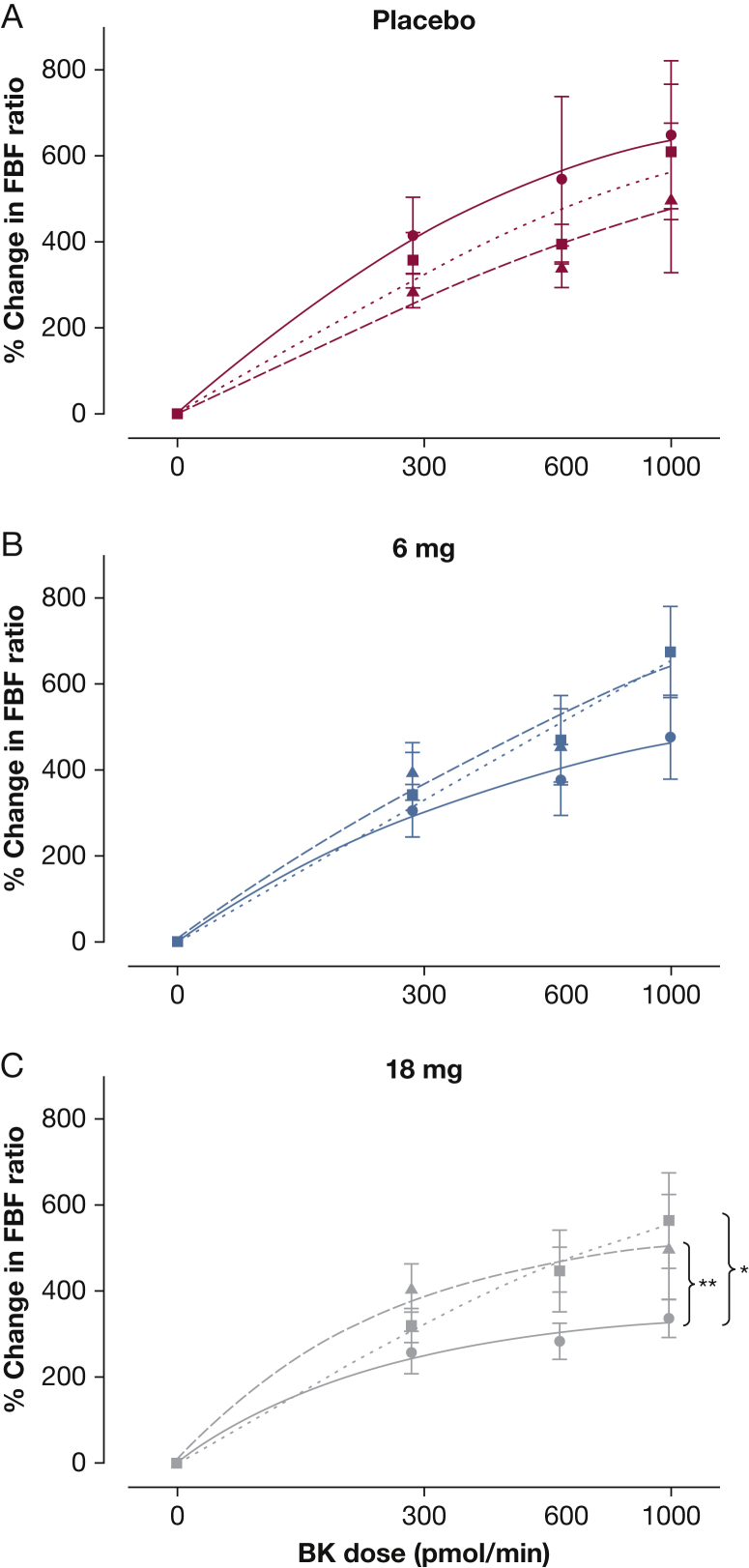
Phase I clinical trial. Responses to bradykinin in overweight smokers who received (A) placebo, (B) 6 mg, and (C) 18 mg of active drug. Bradykinin induced significant vasodilation on all 3 days in all three treatment groups (*P* < .0001). Forearm blood flow improved overall in the active drug group (*P* = .007), with the greatest effect in the 18-mg active drug group, after acute dosing (*P = .02 in C) and after 14 days chronic dosing (**P = .003 in C). Solid lines represent predose; small dotted lines represent acute dose, and long dotted lines represent chronic dose. See Figure 2 legend for expansion of abbreviations.

In a post hoc analysis of the forearm blood flow ratio, there was an improvement in bradykinin-induced responses following dosing with the active drug compared with placebo (*P* = .007), with the greatest effect in the active-drug 18-mg group. In this group, the maximum bradykinin response improved from 338% ± 46% before dosing to 566% ± 110% after a single dose (*P* = .02) and to 503% ± 123% after chronic dosing (*P* = .003) (Fig 5).

LNMMA and aspirin inhibited basal flow equally on all 3 days in the three treatment arms (e-Table 1). BP remained stable, and there were no changes to tPA in response to BK or in PAI-1 release (data not shown).

## Discussion

The findings from these studies suggest that COPD and smoking are associated with impaired overall endothelial function and reduced stimulated vascular EET production. Proof-of-mechanism data demonstrate that sEH inhibition with GSK2256294 results in improvements in vascular function both in vitro and in vivo.

We elected to study patients with COPD and overweight smokers, as the mechanisms behind COPD, smoking, and cardiovascular disease remain poorly understood. Both smokers and patients with COPD exhibit low-grade systemic inflammation,^1^ which plays a key role in endothelial activation, resulting in endothelial dysfunction and the initiation of atherosclerosis.^23^ It has been demonstrated that patients with COPD,^5^ smokers,^24^ and ex-smokers^25^ exhibit a similar degree of endothelial dysfunction, suggesting that smoking may be the key contributing factor. Cardiovascular risk factors are more likely to cluster in obesity, manifesting as a syndrome of increased adipocytes, hyperglycemia, and dyslipidemia, with underlying low-grade inflammation. In normotensive overweight subjects with metabolic syndrome, acetylcholine-induced rather than bradykinin-induced vasodilation is reduced, possibly suggesting a lesser degree of endothelial dysfunction.^9^ However, the extent to which EETs contributed to this endothelial dysfunction remained unclear. Our study was the first to interrogate this further, and forearm blood flow data suggest that EET production is impaired similarly in patients with COPD and overweight smokers, supported by plasma quantification of EET/DHET as a representative of sEH activity.

We observed a trend toward reduced baseline EET/DHET in patients with COPD and overweight smokers, and when comparing the two matched control groups, the baseline EET/DHET ratio was slightly less in the younger matched control subjects for overweight smokers (matched control group 2) than those for COPD (matched control group 1). However, human plasma EET and DHET levels are notoriously difficult to quantify due to their instability; thus, definitive conclusions cannot be drawn from these insignificant results but can only be taken in context of our forearm blood flow data and previous published data. In animals, obesity is associated with reduced hepatic expression of EET-producing CYP2C enzymes.^26^ In mesenteric arteries of obese Zucker rats, there are reduced CYP2C and CYP2J enzymes, with enhanced activity of sEH enzymes.^27^ Increased sEH activity may represent more advanced inflammation, as in coronary artery disease; those who are obese or who smoke exhibit the lowest EET/DHET ratio.^15^ sEH activity is associated with forearm blood flow, as subjects with the Lys55Arg polymorphism in the sEH encoding gene (*EPHX2*) exhibit higher sEH activity and reduced vasodilator responses to bradykinin.^28^ Smoking can also significantly upregulate *EPHX2*,^29^ and this is associated with increased coronary artery calcification in humans.^13^

The reduced EET synthesis and endothelial dysfunction observed in patients with COPD and overweight smokers may be a result of chronic low-grade inflammation secondary to smoking.^30^ In animals, dimethyl sulfoxide-soluble smoke particles can upregulate endothelium-derived vasoconstrictors through the nuclear factor kappa light-chain enhancer of activated B cells (NF-κB),^31^ a pivotal protein controlling the transcription of genes relevant to the pathophysiology of the blood vessel wall, including adhesion molecules and cytokines.^10^ EETs exert their antiinflammatory effects by inhibiting the activation of NF-κB.^10^ Inflammatory states are associated with downregulation of hepatic and extrahepatic CYP450 enzymes, resulting in a vicious cycle of reduced EET production and an ineffective EET-mediated antiinflammatory effect both locally and systemically.^32^

GSK2256294 is a potent sEH inhibitor that exerts high levels of sEH enzyme inhibition both in vitro^19^ and in vivo.^20^ In human left internal mammary arteries, 11,12-EETs are the most potent regioisomer,^33^ and we confirmed that both 11,12-EET- mediated and bradykinin-mediated vasodilation were enhanced in the presence of GSK2256294 in human resistance arteries. In animal models of cigarette smoking and obesity, sEH inhibition improves lung^34^ and endothelial function^35^ and attenuates pulmonary inflammation, as reflected by reduced inflammatory cells, including neutrophils and macrophages.^19^ In human bronchial cells, treatment with exogenous EETs protects against cigarette smoke extract-induced injury.^36^ Consistent with in vitro results, both acute and chronic sEH inhibition for up to 2 weeks improves responses to bradykinin.

No changes were observed in tPA release following sEH inhibition. tPA is a fibrinolytic serine protease that is released from the endothelium and regulates degradation of intravascular fibrin. Impaired tPA release can be associated with coronary atherosclerosis and cigarette smoking.^25^ Treatment of human endothelial cells with exogenous EETs, particularly 11,12-EETs, can increase tPA protein expression in a dose- and time-dependent manner, possibly due to activation of a G-protein, while not affecting PAI-1, the endogenous inhibitor of tPA.^11^ tPA release may also be dependent on the agonist, and in this group of overweight smokers, substance P may elicit a greater response.^24^

Some limitations of this study warrant consideration. Since the main focus of the phase I clinical trial was on safety, tolerability, and pharmacokinetics of GSK2256294 in healthy volunteers, we were not able to test this novel drug in patients with COPD. In addition, the lack of a nonsmoking control group in the phase I clinical trial means that the magnitudes of the effects of both doses of GSK2256294 were relatively small and similar to the variance in bradykinin responses in the placebo group. Therefore, phase II studies in larger patient groups are required to draw definitive conclusions.

Some evidence also suggests that in NO-deficient conditions, EETs may be upregulated.^9^ Thus, by creating an NO-deficient milieu during the forearm blood flow study with LNMMA, we may have masked any further upregulation of EETs by sEH inhibition. Larger clinical trials in patients with COPD, without concomitant inhibition of NO synthase, would be required to further understand the clinical impact of sEH inhibition. This must also be approached with caution because of the potential of EETs to stimulate angiogenesis, and possibly modulate cancer genesis and metastasis,^37^ although, interestingly, dual-action cyclooxygenase and sEH inhibition may in fact suppress cancer.^38^ We found no changes in serum vascular endothelial growth factor, the active drug group with this dosing regimen, after 14 days.^20^

## Conclusions

Patients with COPD and overweight smokers have impaired endothelial function and dysregulated EETs signaling. sEH inhibition can augment bradykinin-induced vasodilation in human resistance vessels both in vitro and in vivo, suggesting that sEH inhibition may be a novel therapeutic target to ameliorate cardiovascular risk in patients with smoking-related endothelial dysfunction.

## Figures and Tables

**Table 1:** Demographics for All Study Subjects

Subject Demographics (mean ± SD)	COPD Group			Overweight Smoker Group			Phase I Clinical Group (Overweight Smokers)			
	COPD (n = 12)	Control (n = 12)	*P* Value	Overweight Smokers (n = 11)	Control Group (n = 12)	*P* Value	6-mg Dose (n = 11)	18-mg Dose (n = 11)	Placebo (n = 6)	*P* Value
Age, y	63 ± 6	64 ± 7	.70	48 ± 8	49 ± 10	.47	43 ± 10	42 ± 9	41 ± 8	.92
BMI, kg/m^2^	27 ± 3	26 ± 3	.33	30 ± 3	25 ± 2	.0001	31 ± 2	31 ± 2	31 ± 3	.72
Height, m	1.75 ± 0.3	1.77 ± 0.1	.33	1.82 ± 0.1	1.80 ± 0.1	.73	1.83 ± 0.06	1.78 ± 0.04	1.76 ± 0.09	.11
Weight, kg	84 ± 12	82 ± 11	.77	103 ± 13	80 ± 7	.0001	103 ± 10	98 ± 10	95 ± 9	.25
Supine SBP, mm Hg	130 ± 17	133 ± 7	.52	130 ± 14	124 ± 14	.26	128 ± 16	141 ± 13	130 ± 13	.09
Supine DBP, mm Hg	80 ± 3	82 ± 5	.77	82 ± 5	78 ± 8	.34	79 ± 7	82 ± 8	76 ± 5	.26
Pack-years	39 ± 17	0	NA	21 ± 11	0	NA	20 ± 10	17 ± 9	16 ± 6	.58

Subject demographics for the physiological study and phase I clinical trial. There were no significant differences in the demographics between overweight smokers and healthy matched control group in the physiological study and no differences between placebo, 6 mg, and 18 mg active drug. For the physiological study, the *P* value was calculated using a Student *t* test for COPD vs the matched control groups, and for the phase I clinical trial, the *P* value was calculated using one-way analysis of variance comparison between three treatment groups.

DBP = diastolic BP; NA = not available; SBP = systolic BP.

## Supplementary Data

e-Online Data
