# Supplementary material for: Mechanisms of Vascular Dysfunction in COPD and Effects of a Novel Soluble Epoxide Hydrolase Inhibitor in Smokers
Source: Chest. 2017 Mar;151(3):555–63. doi: 10.1016/j.chest.2016.10.058 (PMC5332206; doi:10.1016/j.chest.2016.10.058)
Supplement: e-Online Data [file mmc1.pdf]

CHEST Online Supplement

Mechanisms of Vascular Dysfunction in COPD and Effects of a Novel Soluble Epoxide Hydrolase Inhibitor in Smokers

Lucy Yang, MBChB; Joseph Cheriyan, MBChB; David D. Gutterman, MD; Ruth J. Mayer, PhD; Zsuzsanna Ament, PhD; Jules L. Griffin, PhD; Aili L. Lazaar, MD; David E. Newby, PhD; Ruth Tal-Singer, PhD; and Ian B. Wilkinson, DM

CHEST 2017; 151(3):555-563

Online supplements are not copyedited prior to posting and the author(s) take full responsibility for the accuracy of all data.

© 2016 AMERICAN COLLEGE OF CHEST PHYSICIANS. Reproduction of this article is prohibited without written permission from the American College of Chest Physicians. See online for more details. **DOI:** 10.1016/j.chest.2016.10.058

e-Appendix 1. Materials and Methods

Plasma quantification of EETs/DHETs

Venous blood samples were collected from the same arm as the arterial infusion at baseline, and before and after each dose of bradykinin. Approximately 2 mL of blood was collected in lithium heparin tubes, immediately centrifuged at 4°C at 4600 rpm for 5 minutes (Hettich Zentrifugen Rotanta 460R, Germany), then rapidly frozen with liquid nitrogen or dry ice, and stored in -80°C. Thawed samples were prepared by methanol crash method,[1] and EETs and DHETs were measured by high performance liquid chromatography and tandem mass spectrometry using an Aquity Ultra Performance Liquid Chromatogram (Waters Ltd., Manchester) coupled with an AB Sciex 5500 mass spectrometer (Ab Sciex, Warrington).[2]

Effect of GSK2256294 *in vitro*

For *in vitro* assessment of human microvascular function, tissue samples were obtained from otherwise discarded visceral fat (omentum and mesentery) of patients undergoing surgeries, including colectomy, renal transplantation, liver resection, and breast reconstruction. Comorbid conditions included hypertension, cancer, and inflammatory bowel disease, and diverticulitis, but samples were taken from unaffected areas wherever possible. Medication history was unavailable, but tissue was washed in buffer for approximately one hour prior to study, in order to minimise the effects of any concomitant drugs.

Human resistance arteries (100–200 μm) were isolated from the discarded surgical tissue, and cannulated with two glass micropipettes for videomicroscopic measurements of diameter.[3] Vessels were pressurised to an intramural pressure of 60 mmHg under no-flow conditions and equilibrated at 37°C in Krebs-PSS gassed with 21% O_2-5% CO_2. Arteries were pre-constricted with endothelin-1 (ET-1; 0.3 nM) to 50%, and the bradykinin response was examined before and after 30min of L-nitroarginine methyl ester (L-NAME; 100 μM) and indomethacin (10 μM). Dose response curves to bradykinin (BK,10^{-10} to 10^{-4} M), endothelin-1 (ET-1, 30 to 900 pM), 8,9-EET (10^{-10} to 10^{-6} M) and 11,12-EET (10^{-10} to 10^{-6} M) were generated in the presence or absence of GSK2256294. Responses to papaverine (10^{-4} M) were determined at the end. Vascular responses were expressed as percent maximal relaxation to ET-1 preconstriction; 100% represents the passive baseline diameter.

Statistical Analysis

FBF data were analysed using LabChart 8 (ADInstruments, Oxford, United Kingdom), and statistical analysis was performed in R version 3.2.2 and GraphPad Prism (Version 6, GraphPad Software, California, USA). To test the hypothesis that EETs modulate basal tone, baseline FBF change in response to inhibitors were analysed as change in the infused arm only to eliminate possible systemic effects, and compared using paired Student’s *t*-test or one-way analysis of variance (ANOVA). Agonist responses were analysed

Online supplements are not copyedited prior to posting and the author(s) take full responsibility for the accuracy of all data.

as forearm blood flow ratio (infused/non-infused arm), adjusted for baseline, and log-transformed. To assess EETs mediated agonist induced response in COPD and healthy subjects, log-transformed ratio increase were compared using repeated measures ANOVA with a nested three-way interaction term for multiple comparisons between the disease groups (COPD vs. healthy), and within subject comparisons between three bradykinin doses (100, 300 and 1000 pmol/min), and in the presence of fluconazole. Detailed *post-hoc* comparisons were made with Tukey adjustment.

Plasma concentrations of each regio-isomer of the eicosanoids were corrected for baseline (concentration/baseline), and expressed as EET/DHET ratio as a surrogate of sEH activity, and presented as sum of EET/DHETs ratio.[4] Sum of corrected ratios were log transformed, and analysed by repeated measures ANOVA with a nested three-way interaction term for multiple comparisons between groups (COPD vs. healthy), and within subject comparisons in the response to bradykinin, and bradykinin plus fluconazole.

To assess the effects of GSK2256294, *in vitro* studies of arteriolar reactivity was analysed with a one-way repeated measures ANOVA for dose and condition, with a Tukey *post-hoc* correction. When vessels from the same patient were used for identical protocols, values were averaged for statistical analysis ("n's" represent the number of subjects, not vessels). ED50's were compared by *t*-test in the presence or absence of inhibitor. *In vivo*, changes in FBF in the infused arm in response to bradykinin in acute and chronic dosing was compared to pre-dosing. All three treatment arms (placebo, 6 mg, and 18 mg) were fitted in the same model with three agonist doses (change from baseline flow in response to 300, 600 and 1000 pmol/min bradykinin) for each visit (Pre-dose, Day 1, and Day 14) via a three-way interaction term. A *post-hoc* analysis of the FBF ratio (infused/non-infused) was performed to adjust for any changes in the non-infused arm. Log-transformed baseline adjusted ratios were compared using repeated measures ANOVA with a nested three-way interaction term for multiple comparisons between the three treatment arms (placebo, 6 mg and 18 mg active drug), and within subject comparisons between bradykinin doses and treatment days, with Tukey adjustment. A similar analysis was performed for SNP responses. Results are expressed as mean±SEM unless otherwise indicated.

Online supplements are not copyedited prior to posting and the author(s) take full responsibility for the accuracy of all data.

e-Table 1. Phase 1 clinical trial: percentage inhibition of basal flow in the infused arm by LNMMA plus aspirin in the placebo, 6mg and 18mg groups.

	Percentage inhibition of baseline flow in the infused arm by LNMMA and aspirin (Mean ± SEM)					
	Placebo	Inhibitor effect	6mg	Inhibitor effect	18mg	Inhibitor effect
**Pre-dosing**	-35.46±5.65	p=0.01	-31.21±5.35	p=0.003	-30.53±6.23	p=0.004
**Day 1 post-dosing**	-22.22±4.56	p=0.24	-19.14±6.72	p=0.07	-24.72±4.79	p=0.0008
**Day 14 post-dosing**	17.67±9.88	p=0.59	-21.40±9.33	p=0.03	-28.18±4.91	p=0.02
**Difference between pre-dosing and Day 1**	p=0.98		p=1.00		p=0.67	
**Difference between pre-dosing and Day 14**	p=0.99		p=0.99		p=0.44	

e-Table 1. Percentage change in baseline forearm blood flow in the infused arm following LNMMA plus aspirin at pre-dose, Day 1, and Day 14 post-dose. Overall, LNMMA and aspirin had a significant inhibitory effect (p<0.0001), but this effect is variable, although no different between treatment days. There were no differences in inhibition between placebo, 6 mg or 18 mg (p=0.33). Abbreviations: LNMMA; N^G-monomethyl-L-arginine, SEM; standard error of the mean.

References:

1. Puri P, Wiest MM, Cheung O, et al. The plasma lipidomic signature of nonalcoholic steatohepatitis. *Hepatology* 2009;50(6):1827–1838.

2. Hall Z, Ament Z, Wilson CH, et al. Myc expression drives aberrant lipid metabolism in lung cancer. *Cancer Res* 2016;canres.3403.2015.

3. Miura H, Wachtel RE, Liu Y, et al. Flow-induced dilation of human coronary arterioles : important role of Ca2+-activated K+ channels. *Circulation* 2001;103(15):1992–1998.

4. Schuck RN, Theken KN, Edin ML, et al. Cytochrome P450-derived eicosanoids and vascular dysfunction in coronary artery disease patients. *Atherosclerosis* 2013;227(2):442–8.

e-Figure 1.

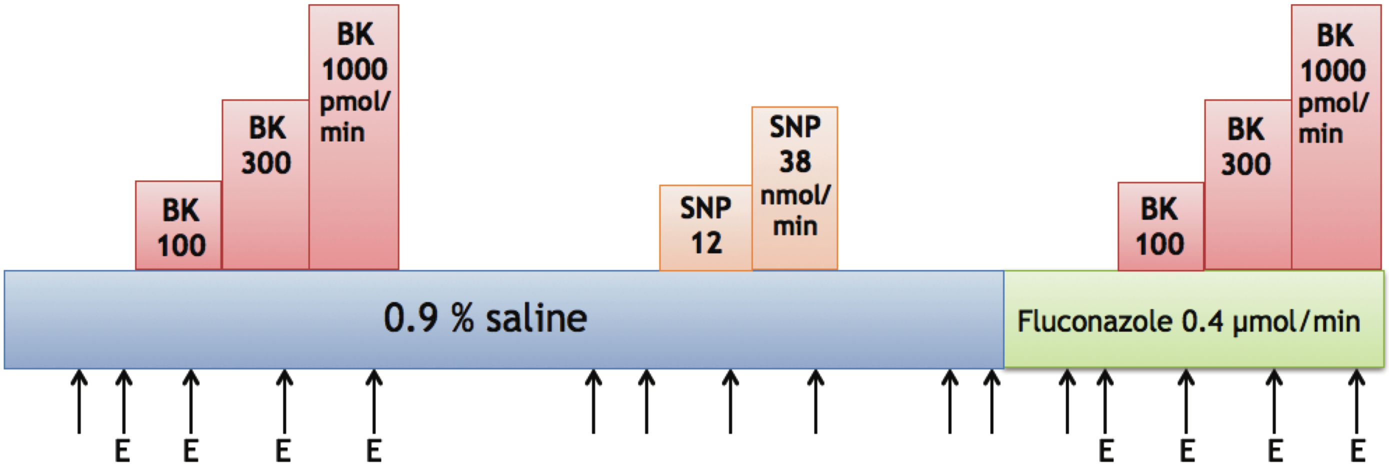

e-Figure 2.

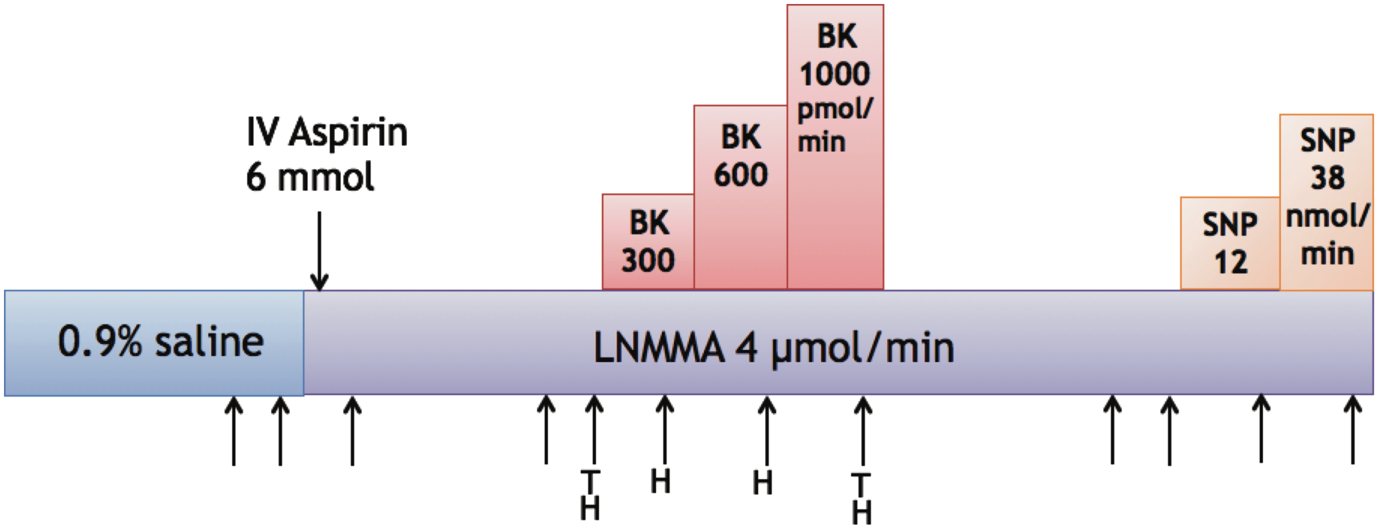

161747
